# Federated Learning with Pareto Optimality for Resource Efficiency and Fast Model Convergence in Mobile Environments †

**DOI:** 10.3390/s24082476

**Published:** 2024-04-12

**Authors:** June-Pyo Jung, Young-Bae Ko, Sung-Hwa Lim

**Affiliations:** 1 Department of AI Convergence Network, Ajou Univeristy, 206, World Cup-ro, Suwon-si 16499, Republic of Korea; forjune@ajou.ac.kr; 2 Department of Multimedia, Namseoul University, 91, Daehak-ro, Cheonan-si 31020, Republic of Korea; sunghwa@nsu.ac.kr

**Keywords:** federated learning, Pareto optimality, mobile communication

## Abstract

Federated learning (FL) is an emerging distributed learning technique through which models can be trained using the data collected by user devices in resource-constrained situations while protecting user privacy. However, FL has three main limitations: First, the parameter server (PS), which aggregates the local models that are trained using local user data, is typically far from users. The large distance may burden the path links between the PS and local nodes, thereby increasing the consumption of the network and computing resources. Second, user device resources are limited, but this aspect is not considered in the training of the local model and transmission of the model parameters. Third, the PS-side links tend to become highly loaded as the number of participating clients increases. The links become congested owing to the large size of model parameters. In this study, we propose a resource-efficient FL scheme. We follow the Pareto optimality concept with the biased client selection to limit client participation, thereby ensuring efficient resource consumption and rapid model convergence. In addition, we propose a hierarchical structure with location-based clustering for device-to-device communication using k-means clustering. Simulation results show that with *prate* at 0.75, the proposed scheme effectively reduced transmitted and received network traffic by 75.89% and 78.77%, respectively, compared to the FedAvg method. It also achieves faster model convergence compared to other FL mechanisms, such as FedAvg and D2D-FedAvg.

## 1. Introduction

The accelerated development of big data has resulted in the increasing application of artificial intelligence (AI) technologies. The International Data Corporation has predicted that the amount of data generated through Internet of things (IoT) devices will reach 79.4 ZB in 2025 [1], exceeding the capacities of IoT and mobile devices worldwide [2]. Most of the data generated by a device are processed locally or in a remote cloud server. However, this process involves three main problems pertaining to the constraints associated with real environments and remote cloud servers [3]:

1. Network Congestion: network congestion on a remote cloud server occurs when numerous user devices simultaneously send data to the server.
2. Privacy Leak: private user experience data may be leaked due to malicious network attacks during transmission.
3. Resource Constraints: the capacity of network resources (e.g., wireless channel subcarriers and bandwidth) and user devices (e.g., computing performances and battery life) is limited.

Mobile edge computing (MEC) has emerged as a solution to these problems because it can process and store the big data generated by devices. MEC divides traffic and computational processes from a remote cloud server to an edge server, thereby reducing the distance between the server and clients. Specifically, instead of directly sending all the data to a remote cloud server for processing and storage, MEC analyzes, processes, and stores data at an edge server. MEC can reduce the latency and real-time processing time for high-bandwidth applications and even eliminate certain resource constraints associated with client devices. In addition, as the big data generated by devices can be used by various AI applications (e.g., autonomous driving, medical tests, and recommendation systems), machine learning (ML) tasks constitute the major workload in MEC [4].

However, MEC cannot solve the privacy leak problem because the personal data obtained from user devices are stored or processed on an edge server. Furthermore, the network congestion issue is not resolved because large amounts of data are transmitted for ML tasks. In this context, federated learning (FL) has attracted attention as a distributed learning method to perform training over large amounts of generated data and update models on local nodes (e.g., mobile devices). In this manner, FL can alleviate network congestion, prevent privacy leaks, and reduce resource consumption for computation and communication [5]. Furthermore, the integration of FL with MEC will be a pivotal step towards achieving ubiquitous intelligence in 6G networks. This combination will enable more efficient utilization of the vast amounts of data generated by devices through MEC [6]. Notably, in FL, the size of model parameters updated by training local devices, which may be billions in number, can reach tens of megabytes [7]. Consequently, a bottleneck may occur during the aggregation of model parameters in a parameter server (PS). These bottlenecks may be exacerbated by conventional FL frameworks as they are based on direct communication between clients and a PS. Consequently, it is difficult to achieve model convergence because of the error in transmitting model parameters. This aspect adversely affects the model scalability, and thus, more communication rounds and local training are required to optimize the model [8].

Device-to-device (D2D) communication is a localized version of peer-to-peer communication that enables direct access among local devices without base stations or access points. This framework effectively reduces communication resource consumption and network delay through the use of short-distance wireless communication and increases the coverage of systems [9]. D2D communication can overcome the above-mentioned problems because it has a hierarchical structure in the FL architecture and can decrease the communication distance between mobile devices and a PS, thereby optimizing the consumption of communication resources.

The preliminary version of this study was presented as a conference paper [10]. We proposed an FL framework with a hierarchical structure, in which the model parameters of local nodes in a cluster are aggregated to a leader client (LC), and the LCs send the aggregated model parameters to a PS. Considering the potential of D2D communication, we developed an FL mechanism to exploit the benefits of resource consumption and short-distance communication delay. Clusters among nodes were generated via k-means clustering. In the clusters formed by k-means clustering, clients communicate with each other within a predefined threshold distance for D2D communications, and only a subset of these clients participate in FL. In this paper, We used the Pareto principle to show that the participation of a small number of clients according to a biased criterion can improve model convergence and alleviate the bottleneck in aggregating model parameters. Enhancing the preliminary version, in this study, Pareto optimality is newly employed to ensure reasonable client selection by exploiting the client resource states and training losses. Moreover, we have added credibility through additional experiments in this paper. The main contributions of this study can be summarized as follows:

- We propose an FL mechanism with a hierarchical D2D structure by clustering clients on the basis of the location and communication range of each client. This mechanism can effectively reduce the wireless communication traffic generated when the FL model is updated for each client.
- We propose a biased client-selection method for a clustered structure by using Pareto optimality. This client-selection method employs high training loss values to accelerate model convergence and reduce resource consumption.

## 2. Related Work

FL is an alternative distributed ML method. FL differs from conventional distributed ML in that it involves an extremely large number of clients with heterogeneous and unbalanced local data distributions. A key task of FL is the generation of learning models using the data collected from clients. These data are stored in local devices, which can help prevent privacy leaks and avoid model divergence due to insufficient data and failure to participate owing to a lack of resources (e.g., wireless channel subcarriers, bandwidth, computing performance, and battery life). The convergence of a learning model and resource consumption in FL exhibit a trade-off relationship. Thus, many researchers have attempted to improve the efficiency of FL by simultaneously optimizing its performance aspects.

Federated averaging (FedAvg) [11], which is the most conventional FL mechanism, adjusts the batch size and epochs of federated stochastic gradient descent to average the gradient descent generated from a learning process, thereby significantly reducing the overall number of communication rounds by iterating more local updates on a client device. FedAsync [12] is an asynchronous FL mechanism for updating global models, in which the mixing weight is adaptively set as a function of staleness. Notably, in [11,12], experiments were conducted on non-iid data, i.e., data that are not independently and identically distributed. However, a theoretical guarantee could not be realized in a convex optimization setting. In [13], convergence guarantee for FedAvg was ensured without the impractical assumptions that the data are iid and all clients are available.

A large number of participating clients in FL may lead to server-side congestion and bottlenecks in aggregating client model parameters. Additionally, a large number of participating clients can affect the model convergence for non-iid data [13]. By appropriately selecting the participating clients, the above-mentioned problems can be solved, and model convergence can be improved. Unlike FedAvg, in which clients are randomly selected, certain researchers [14] considered clients with high loss values and proved that biased client selection is directly related to model convergence. The FedCS FL protocol was developed [15] for selecting clients within a deadline to manage the resources of heterogeneous clients. This method employed biased client selection; however, it did not ensure the convergence of models for non-iid and heterogeneous data. Furthermore, stragglers may be present in a mobile communication or IoT environment, which cannot participate in FL because the network connection is not persistent or a client device has shut down. The presence of stragglers may hinder model convergence. Therefore, the FLANP FL framework was proposed [16] to alleviate the effect of stragglers by adaptively selecting clients in different communication rounds according to their computation speeds.

FL mechanisms with various structures have been proposed. In the hierarchical FL (HFL) mechanism proposed in [17,18,19], a client and server communicated through an intermediate medium rather than a direct communication structure. In [19], the hierarchical edge federated learning (HED-FL) model enhances traditional FL with a multi-layered edge node architecture for energy-efficient learning. Two heuristic methods were also introduced to assess the effects of static and dynamic round execution across these layers. Moreover, a hierarchical cluster-based structure was developed [17], which divided clients into several clusters based on resource constraints. A leader node (LN) was elected, which was similar to an intermediate server. Only the LN directly communicated with a PS. Thus, the bottleneck that may have occurred in the PS was eliminated, and the consumption of communication resources was reduced. Similarly, an edge server was deployed between a PS and the clients [18]. The edge association problem was solved using an evolutionary game between the clients and the edge server. The communication resource allocation problem between the edge server and PS was solved using a Stackelberg differential game.

D2D and peer-to-peer (P2P) communication has been introduced to reduce the communication overhead for the efficient transmission of model parameters. Certain researchers [20] examined a social attribute that was used for k-means clustering in D2D communication. A software-defined networking controller was used for clustering by calculating the social attributes between devices, rather than clustering with an unspecified majority. Other researchers proposed algorithms for D2D communication with resource allocation [21], which could efficiently manage resources and interference. Zhang et al. [22] proposed a D2D-assisted hierarchical FL scheme to reduce the communication overhead in D2D environments. Semi-decentralized federated edge learning (SD-FEEL) [23] proposes a structure that aggregates clients’ model parameters and exchanges model parameters with neighboring edge servers, followed by broadcasting the updated models. Two timescale hybrid FL (TT-HF) [24] extends the FL architecture through aperiodic local and global model consensus procedures based on D2D communications, proposing a new model of gradient diversity and an adaptive control algorithm. In another framework [25], clients communicated with one another without a server for aggregating model parameters in FL. Moreover, topology construction was conducted through deep reinforcement learning for P2P FL [26].

The novelty of our framework lies in the following aspects: In [22,24,25], they propose FL utilizing D2D communication, and [23] forms clusters for aggregating clients’ model parameters, similar to our work. However, we have introduced the k-means clustering technique for the formation of D2D communication networks. This approach enables the selection of leader clients located in optimal positions without exceeding the communication distance threshold. By collecting and transmitting model parameters within clusters, it offers an ideal solution to alleviate server-side bottleneck issues. In [27], Min-Max Pareto optimization was used to manage the trade-off relationship between the algorithmic fairness and performance inconsistency for each client. FedMGDA+ [28], which is similar to the framework proposed in [27], realizes the multi-objective optimization of robustness, fairness, and accuracy through the Pareto stationary solution. In contrast, we consider that the model performance is proportional to the client’s resource consumption. Therefore, we solve the target problem by using the Pareto optimality and considering the trade-off relationship between the model convergence and resource consumption. In this manner, the proposed method is different from those described in [27,28]: A comparative analysis of FL methods, including FedPO, is encapsulated in Table 1, which delineates the distinct communication method, hierarchical architecture, and client selection strategies employed by each technique.

## 3. Preliminaries

### 3.1. A Brief Overview on Federated Learning

FL performs ML from a federation of clients. The clients train a model using their data and update the training model using gradient descent. After training, the updated model parameters are sent to a PS. The PS updates a global model by aggregating the model parameters trained by each client and calculating the weighted averages according to the number of data samples of each client. The model is defined as the loss function generated by model parameter vector *w* as $f_{i}\left(x_{i},y_{i},w\right)$, where *i* denotes the input data, $x_{i}$ is a feature, and $y_{i}$ is a label. Considering the FL framework with *K* clients, we define $D_{k}$ as the local data sample for client *k*. Then, the loss function for each client on the local dataset can be expressed as in Equation (1), where $N_{k}$ is the size of $D_{k}$ ($N_{k}=\left|D_{k}\right|$).

(1) $$F\left(w\right)=\frac{1}{\sum_{k=1}^{K}N_{k}}\sum_{k=1}^{K}\sum_{x_{i},y_{i}\in D_{k}}f_{i}\left(x_{i},y_{i},w\right)$$

Client *k* updates its gradient descent for each local learning during round *T*, where the learning rate is η>0. The local model parameter in round *t* is $w_{k}^{t}$, defined in Equation (2).

(2) $$g\left(w_{k}^{t}\right)=\frac{1}{N_{k}}\sum_{x_{i},y_{i}\in D_{k}}\nabla f\left(x_{i},y_{i},w_{k}^{t}\right),w_{k}^{t}=w_{k}^{t-1}-\eta \cdot g\left(w_{k}^{t-1}\right)$$

PS aggregates $w_{k}^{t}$ for certain clients, and the weighted averages used to update the global model are

(3) $$w_{ps}^{t+1}=w_{ps}^{t}-\frac{1}{N_{K}}\sum_{k=1}^{K}N_{k}\cdot w_{k}^{t}$$

The goal is to find a model parameter that can converge for all clients and minimize the loss function:

(4) $$w^{*}=\underset{w}{\mathrm{argmin}}\left(F\left(w\right)\right)$$

### 3.2. Pareto Principle and Pareto Optimality

The Pareto principle, named after Italian economist Vilfredo Pareto, posits that 80% of all outcomes result from 20% of the causes. Originally observed in the early 20th century to describe the unequal distribution of wealth in Italy—where 20% of the population owned 80% of the land. In business, it is often used to focus on the most profitable products or the most engaged customers;

In the context of FL, we leverage this principle to allow a smaller but more crucial subset of clients to make significant contributions to the model’s convergence while also alleviating some communication issues. This principle has shown us that not all clients contribute equally, but it does not offer guidance on how to strike a balance between various factors such as computational power, data quality, and contribution to model accuracy.

That is where the concept of Pareto optimality, which asserts that a society achieves maximum satisfaction when no individual can be made better off without making another individual worse off [29], comes into play. In contrast to the Pareto principle, which highlights inequality in contributions, Pareto optimality provides a framework for making trade-offs among competing objectives.

For instance, focusing solely on clients with high computational power could expedite the model’s convergence but at the cost of underrepresenting clients with lower resources, thereby creating a biased model. Pareto optimality seeks to mitigate this by identifying a set of clients that provides the most balanced trade-off between model accuracy and representational fairness.

#### 3.2.1. Basic Definition

The concept of Pareto optimality in a multi-objective optimization context refers to a state being considered Pareto optimal if no other feasible states exist that improve at least one objective without worsening any of the other objectives. Mathematically, let *A* be a set of *n*-dimensional vectors representing possible states. A vector a∈A is considered Pareto optimal if there does not exist any vector b∈A that dominates *a*. Formally, the definition is as follows:

$$∄b\in A\;s.t.\;b_{i}\geq a_{i}\;\forall i,\;\mathrm{and}\;b_{i}>a_{i}\;\mathrm{for}\;\mathrm{at}\;\mathrm{least}\;\mathrm{one}\;i$$

#### 3.2.2. Pareto Front

The Pareto front comprises all Pareto optimal points in the decision space, serving as an essential reference for decision-makers in multi-objective optimization scenarios. Mathematically, for a set *A* and objective functions $f_{1},f_{2},\ldots ,f_{k}$, a point *a* belongs to the Pareto front if:

$$\forall b\in A,∄c\in A\;s.t.\;f_{i}\left(c\right)\geq f_{i}\left(b\right)\;\forall i,\;\mathrm{and}\;f_{j}\left(c\right)>f_{j}\left(b\right)\;\mathrm{for}\;\mathrm{at}\;\mathrm{least}\;\mathrm{one}\;j$$

Here, $f_{i}$ and $f_{j}$ are specific objectives among the *k* different objectives under consideration. This ensures that each point on the Pareto front is not dominated by any other point across all objectives [30].

## 4. FedPO: Federated Learning with Pareto Optimality

### 4.1. Problem Formulation

FedPO is an HFL method that uses biased client selection to identify the participating clients by solving for the Pareto optimality using the training loss and resource state of clients.

In FL, a trade-off relationship exists between the model convergence and resource consumption. More local training or communication rounds for aggregation are required to increase the convergence speed of the model, resulting in the consumption of large amounts of network and computational resources. We use a method [14] that selects clients with a high loss value to accelerate model convergence. Specifically, we select the client with the optimal state of model convergence (high loss value) and resources, following Pareto optimality. We assume a two-dimensional Euclidean space $R^{2}$, ordered by a Pareto cone $R_{+}^{2}$, to solve a Pareto optimality point for resources and loss. In addition, we assume that *E* is a locally convex space, and $C_{E}$ is a convex pointed cone that defines a partial order $\left(\text{≥}C_{E}\right)$ in *E* [29].

**Definition** **1.** *We define* $A=\{\left(\alpha ,\beta \right)\subseteq R^{2}:\alpha \geq 0,\;\beta \geq 0\;$*, where α and β are the resource state and loss value for a client, respectively}*.

*1*. *We assume an elliptic function $\frac{x^{2}}{a^{2}}+\frac{y^{2}}{b^{2}}=1$ to illustrate Pareto optimality*.
*2*. *Point c∈A is an ideal maximum point of A if $\left(\alpha ,\beta \right)\geq C_{E}$ for every (α,β)∈A and closest to the elliptic function*.

In Definition 1, *a* is the highest resource state of the client in set $R=\{r_{1},\ldots ,r_{k}\}$, and the resource states are modeled to have a Gaussian distribution according to [31]. *b* is the server’s training loss value for the model parameters $w_{ps}^{t}$, which are aggregated at time *t*. *a* and *b* can be defined as follows:

(5) $$a=\max(r_{k}^{t})$$

(6) $$b=f(x_{i},y_{i},w_{ps}^{t})$$

We consider the client at point *c* that satisfies Definition 1 as the optimal client for participating in FL.

### 4.2. FedPO Framework

**Figure 1:**
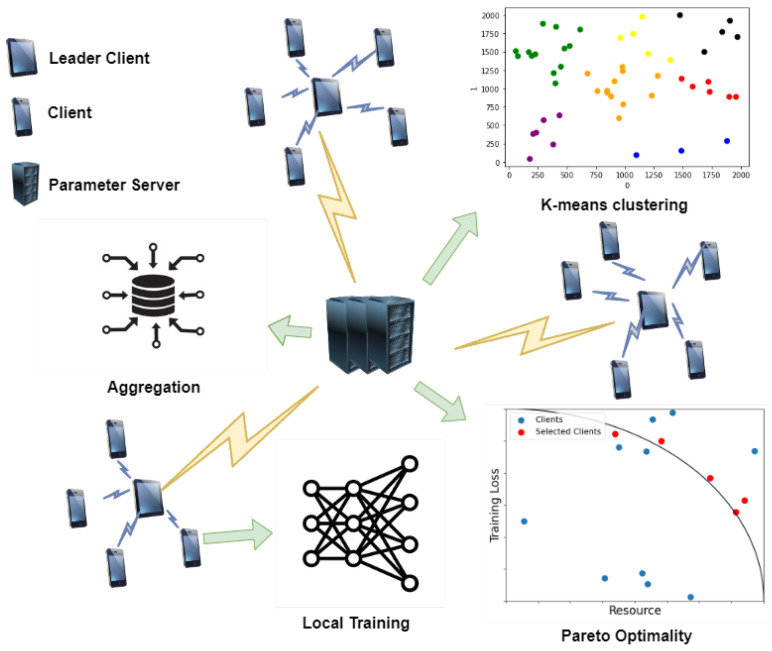
System model.

Figure 1 shows the proposed system model, which consists of clients, LCs, and the PS. The clients are represented by set K={1,…,k}, where *k* is the number of clients. A client updates model parameter $w_{k}$ using the local data generated through user experience. The set of locations for client *K* is represented by $Location=\{l_{1},\ldots ,l_{k}$}. The clusters generated via k-means clustering are expressed as $M=\{M_{1},\ldots ,M_{j}\}$, where *j* is the number of generated clusters and $M_{i}=\left\{x|\;x\;\subseteq K\right\}$. $M_{i}$ is mutually disjoint ($\forall M_{i}\in M,\mathrm{if}\;i\neq j\;\mathrm{then}\;M_{i}\cap M_{j}=Ø$). A *centroid* is a point located at the center of a cluster when *j* clusters are generated, and each centroid in *M* can be represented by $C=\{c_{1},\ldots ,c_{j}\}$. The groups of LCs are expressed as $LeaderClients=\{lc_{1},\ldots ,lc_{j}\}$. In a cluster, the client whose location is the closest to the centroid is selected as an LC, which acts as an intermediate server for HFL as certain clients are selected. Thus, LCs aggregate the updated model parameters of clients and transmit them to the server. The PS updates the global model using the weighted average of the aggregated model parameters, $w_{LC}$, received from all LCs. The PS calculates Pareto optimality to select the clients that will participate in the next round of training for the LCs. In addition, in accordance with the Pareto principle, the proposed method involves fewer clients compared with those in conventional FL, thereby ensuring model convergence for heterogeneous data and the efficient transmission of model parameters. The details of the proposed method are presented in the following text.

**Figure 2:**
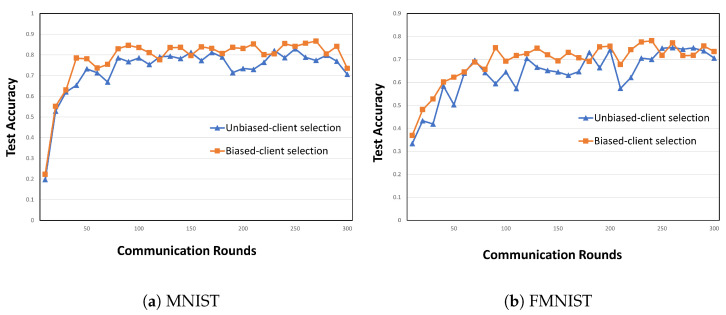
Example of client selection with model accuracy when different methods are applied in LR learning on (**a**) MNIST and (**b**) FashionMNIST in a distributed setting with 20 clients and a participation rate (*prate*) of 0.25.

1. K-means clustering for D2D communication: Compared with short-distance wireless communication [9], D2D communication effectively reduces resource consumption and network delay. We use D2D communication for transmitting the model parameters, training loss, and resource state of clients to the LCs for HFL. The PS builds an intranetwork for D2D communication using k-means clustering, based on the locations of clients. $distortion_{j}$ is the average communication distance between intraclients according to the number of clusters *j*: $Distortion=\{distortion_{1},\ldots ,distortion_{j}\}$, where *j* does not exceed K/2 as the pairing for D2D communication. Additionally, we consider that at least two clients exist in each cluster. Therefore, when the number of clusters is *j*, the average Euclidean communication distance from $lc_{j}$ to the location of each client *k* belonging to cluster $M_{j}$ is expressed as follows:
  (7) $$distortion_{j}=\frac{1}{j}\sum_{i=1}^{j}\sum_{k\in M_{j}}\frac{1}{|M_{i}|}{∥l_{k}-c_{i}∥}^{2}$$
  A *threshold* value is set for the communication distance. The average communication distance from each client in the clusters does not exceed the *threshold*, and the optimal *j* is the maximum value.
  (8) $$j=\underset{j}{\mathrm{argmax}}\left(|M|\right),\;s.t.\;distortion_{j}\leq threshold$$
2. HFL: Similar to [17,22], we regard LCs as intermediate servers. In a cluster, the client located closest to the centroid is selected as the LC to minimize the distance between the LC and other clients in the cluster. The model parameters of the clients belonging to each cluster are transmitted to the LCs. The LCs aggregate the model parameters in round *t* and perform weighted averaging, as follows:
  (9) $$w_{M_{j}}^{t}=\frac{1}{N_{M_{j}}}\sum_{k\in M_{j}}w_{k}^{t}N_{k},\;N_{M_{j}}=\sum_{k\in M_{j}}N_{k}$$
  Thereafter, each LC sends the averaged model parameters to the PS, which aggregates these model parameters. At time *t*, the global model parameters in the PS are $w_{ps}^{t}=\frac{1}{N_{M}}\sum_{i=1}^{j}w_{M_{i}}^{t}N_{M_{i}}$.
3. Biased client selection for Pareto principle and optimality: We use the Pareto principle and optimality to ensure model convergence and to optimize the resource consumption. Figure 2 shows the accuracy of biased and unbiased client-selection methods in FL. On the MNIST and FashionMNIST datasets, biased client selection leads to faster model convergence in the initial stage, and its accuracy is higher than that of unbiased client selection. This result can be interpreted considering the Pareto principle: a small number of clients selected through biased client selection can produce sufficient outcomes. Furthermore, we select clients in accordance with the Pareto optimality function based on two criteria: loss value and resource state. Therefore, according to the convergence analysis in [14], the loss value is adopted as the criterion for using Pareto optimality for client selection. The other criterion is the state of client resources because all clients have finite network and computational resources in actual environments.

### 4.3. Algorithm

The process flow of FedPO is presented as Algorithm 1. In the case of FedAvg, the model parameters must be directly sent to all clients. In contrast, in the proposed method, the model parameters are sent only to the LCs by adopting HFL. In Algorithm 1, *j*, *M*, and $w_{M_{j}}^{t}$ are the number of clusters, set of clusters, and group model parameters of $M_{j}$ in the *t*-th round, respectively. The *LocationBasedClusterting()* procedure in line 3 of Algorithm 1 is presented as Algorithm 2. We build clusters for D2D communication according to client locations using k-means clustering. Algorithm 2 is used to identify the optimal number of clusters and adjust the limit on the communication distance to prevent resource wastage in D2D communication. As the proposed structure is HFL (lines 4 and 5 in Algorithm 1), the model parameters of the PS are transmitted to clients through $lc_{j}$. In addition, $lc_{j}$ transmits the model parameters received by the PS to clients at a low cost using D2D communication [9]. The *SelectClient()* procedure in line 8 in Algorithm 1 selects participating clients (i.e., *SelectedClients*) for FL, which consist of subset *K* presented in Algorithm 3. The PS selects the clients that are closest to the elliptic function through Pareto optimality or whose loss value is larger than that of the PS. The number of participating clients is adjusted by applying prate to each cluster. However, in Algorithm 3, client *k* must be selected if its loss is larger than that of the global model.

**Algorithm 1:** Federated Learning with Pareto Optimality
**Input**:
participation rate prate, random location set of clients location, threshold for the distance between nodes threshold, client set *K*  1:Initialize model $w_{ps}^{0}$  2:**for** Communication round t = 1, 2, …, T **do**  3:   PS performs **LocationBasedClustering**(K)  4:   PS sends global model $w_{ps}^{t}$ to LC  5:   $lc_{j}$ sends $w_{ps}^{t}$ to each client belonging to cluster $M_{j}$ via D2D communication  6:   At this point, $w_{ps}^{t}$ = $w_{M}^{t}$  7:   Each client belonging to cluster $M_{j}$ trains $w_{M_{j}}^{t}$ and updates it using its local data  8:   PS performs **SelectClient**$\left(r_{k}^{t},f\left(x_{i},y_{i},w_{k}^{t}\right),prate\right)$  9:   SelectedClients send $w_{k}^{t}$ to $lc_{j}$10:   $lc_{j}$ aggregates SelectedClients’        $w_{M_{j}}^{t}=\frac{1}{N_{M_{j}}}\sum_{k\in M_{j}}w_{k}^{t}N_{k}$ via D2D communication11:   PS aggregates and updates model        $w_{ps}^{t+1}=\frac{1}{N_{M}}\sum_{i=1}^{j}w_{M_{i}}^{t}N_{M_{i}}$12:
**end for**



**Algorithm 2:** LocationBasedClustering
**Input**:
Client set *K*  1:Clients are clustered based on their location using **k-means clustering**  2:Set of cluster *M* is generated  3:

$distortion_{j}=\frac{1}{j}\sum_{i=1}^{j}\sum_{k\in M_{j}}\frac{1}{|M_{i}|}{∥l_{k}-c_{i}∥}^{2}$

  4:**if** $distortion_{j}\leq threshold$ **then**  5:   $j={\mathrm{argmax}}_{j}\left(|M|\right)$  6:
**end if**
  7:Client closest to the centroid of cluster $M_{j}$ is selected as $lc_{j}$


**Algorithm 3:** SelectClient
**Input**:
Resource state $r_{k}^{t}$, $f(x_{i},y_{i},w_{k}^{t})$, participation fraction prate  1:Clients belonging to $M_{j}$ send loss value $f(x_{i},y_{i},w_{k}^{t})$ and resource state $r_{k}^{t}$ to $lc_{j}$  2:PS selects clients following Pareto optimality with Definition 1  3:**if** $f\left(x_{i},y_{i},w_{k}^{t}\right)\geq f\left(x_{i},y_{i},w_{ps}^{t}\right)$ **then**  4:   $lc_{j}$ receives model parameters from a fraction of clients within |K| * prate based on the high loss value  5:
**end if**
**Output**:

*SelectedClients*



## 5. Performance Evaluation

### 5.1. Simulation Settings

**Environment.** To test the model convergence, we simulate FL using PyTorch and measure the networking overhead using an off-the-shelf network simulation tool named OPNET. We assume a D2D communication network in an area of 2000 × 2000 m. Additionally, we assume that the base station (i.e., PS) is located at the centroid of the network. According to Algorithm 2, all clients in the cluster are located within a one-hop communication distance that does not exceed the threshold. The feasible communication distance (i.e., *threshold*) between clients is referred to from [22,32]. We select FedAvg [11], the most conventional method in FL, which involves aggregating the average of model parameters after multiple local trainings, and D2D-FedAvg [22], the system most similar to ours with features including D2D communication and a hierarchical structure, as our comparison targets. Moreover, we use the simulation parameters specified in [22] to compare the network overhead with those of FedAvg, D2D-FedAvg, and FedPO as detailed in Table 2. For the experiments, *resources* are defined to consist of the computational and communication resources of a client. The distribution of client resources is modeled as a Gaussian distribution, as mentioned previously. We assume that one resource unit is consumed considering the distance over which a client communicates with the PS and the basic resources consumed during the communication and computational processes [11,12].

**Figure 3:**
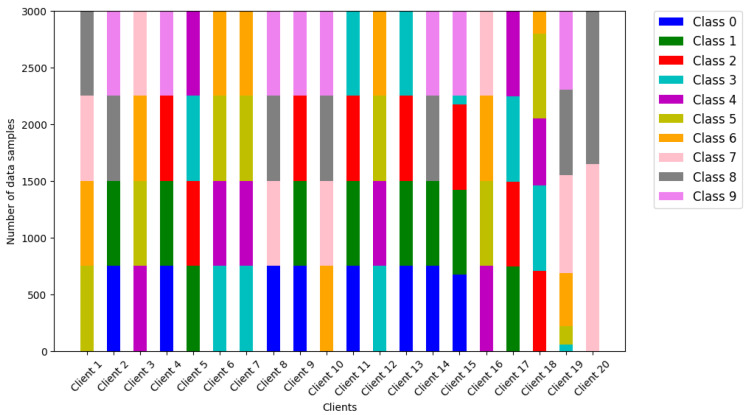
Class per 20 clients and unbalances in MNIST.

**Model and Datasets.** The learning model is tested using logistic regression (LR) and long short-term memory (LSTM), with the training data derived from MNIST and FashionMNIST datasets. Figure 3 shows the configuration using MNIST data, with different classes (typically, four classes) of clients with local data introduced to reflect the non-iid situation in the experiments. Disproportionate amounts of data are assumed to be held by clients, characteristic of non-iid situations.

### 5.2. Simulation Results

**Figure 4:**
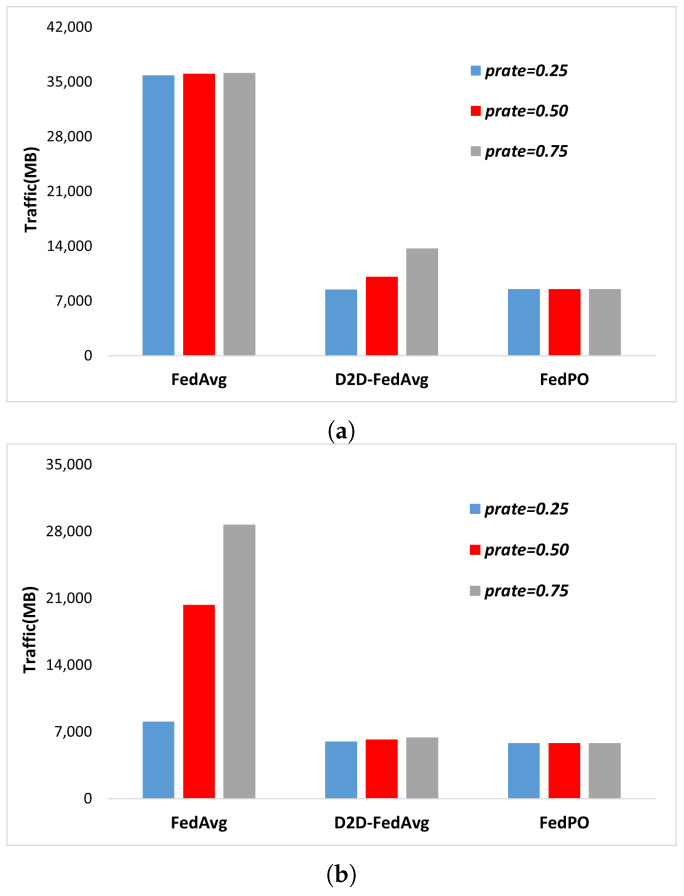
Server-side traffic for different values of *prates*. (**a**) Server-side transmitted traffic for different values of prates. (**b**) Server-side received traffic for different values of prates.

**Resource Efficiency.** The proportion of traffic transmitted and received by the server-side of the trained model is shown in Figure 4a,b, respectively. The number of participating clients is |K|=100, iterations are performed for *T* = 1000 rounds, and the traffic values of FedAvg, D2D-FedAvg, and FedPO are compared. Figure 4a shows that for FedAvg, the transmission traffic is similar for all prate values, because the model parameters are transmitted to all clients in every round. Furthermore, in the case of D2D-FedAvg, as the number of participating clients in FL increases according to prate, residual clients that cannot participate in the grouping process for D2D communication and thus communicate directly with the PS emerge, leading to slightly higher amounts of transmission traffic compared with those of FedPO. This phenomenon occurs because FedPO has a fixed amount of server-side transmission due to the use of location-based k-means clustering for the clients. The same result can be observed in Figure 4b, which represents the amount of traffic received. In the case of FedAvg, the number of clients sending data to the PS increases as prate increases. In contrast, both D2D-FedAvg and FedPO have an intermediate transmitter (e.g., MUE and LC) in the communication process, resulting in less traffic received by the server. This configuration can partially alleviate the potential bottleneck and delay problems, depending on the amount of data received by the PS. The exact ratios corresponding to Figure 4 are listed in Table 3. In terms of the transmitted traffic from the server, the results in Table 3 show that D2D-FedAvg and FedPO have lower overhead than FedAvg, which transmits the parameters to all clients. D2D-FedAvg shows a slight increase in overhead with increasing participation rate, with values of 23.57%, 28.05%, and 37.99%. In contrast, FedPO maintains similar levels of overhead for all participation rates, with values of 23.73%, 23.67%, and 24.11%. In terms of the received traffic from the server, both D2D-FedAvg and FedPO exhibit similar overhead as that of FedAvg with a participation rate of 0.75. These results indicate the superiority of D2D-FedAvg and FedPO.

**Figure 5:**
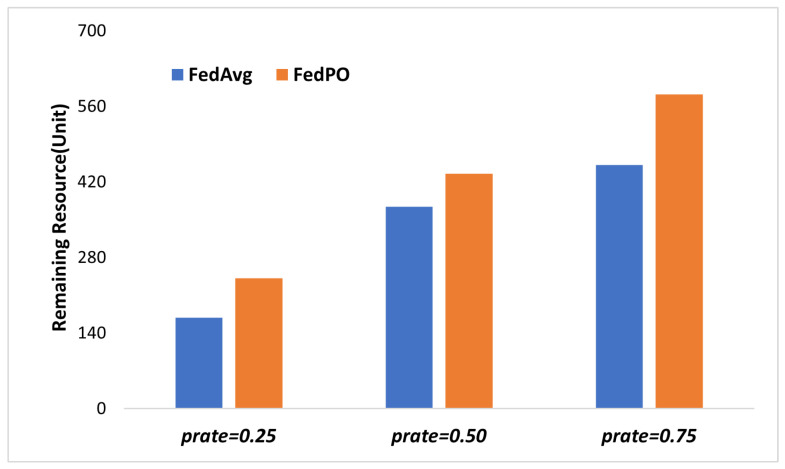
Sum of remaining resources of clients at each round.

In FL, the clients play a crucial role in the learning process as they consume both computational and communication resources. Computational resources are consumed during model training using local data, whereas communication resources are used for model parameter communication. The amounts of resources consumed and remaining for each client after each round represent important criteria for client participation and selection in the next round. To analyze the effect of different FL methods on the resource states of the clients, Figure 5 shows the sum of the remaining resources of clients in each round. We compare FedPO with FedAvg as both D2D-FedAvg and FedAvg have similar algorithms and resource consumption levels. FedPO outperforms FedAvg in terms of the resource conservation of clients. This phenomenon occurs owing to the Pareto optimality-based client selection method used in FedPO, which takes into account the client’s available resources and loss during training. Consequently, FedPO selects those clients who will participate in FL without compromising their resources and model convergence and increases the remaining resources of the clients.

**Figure 6:**
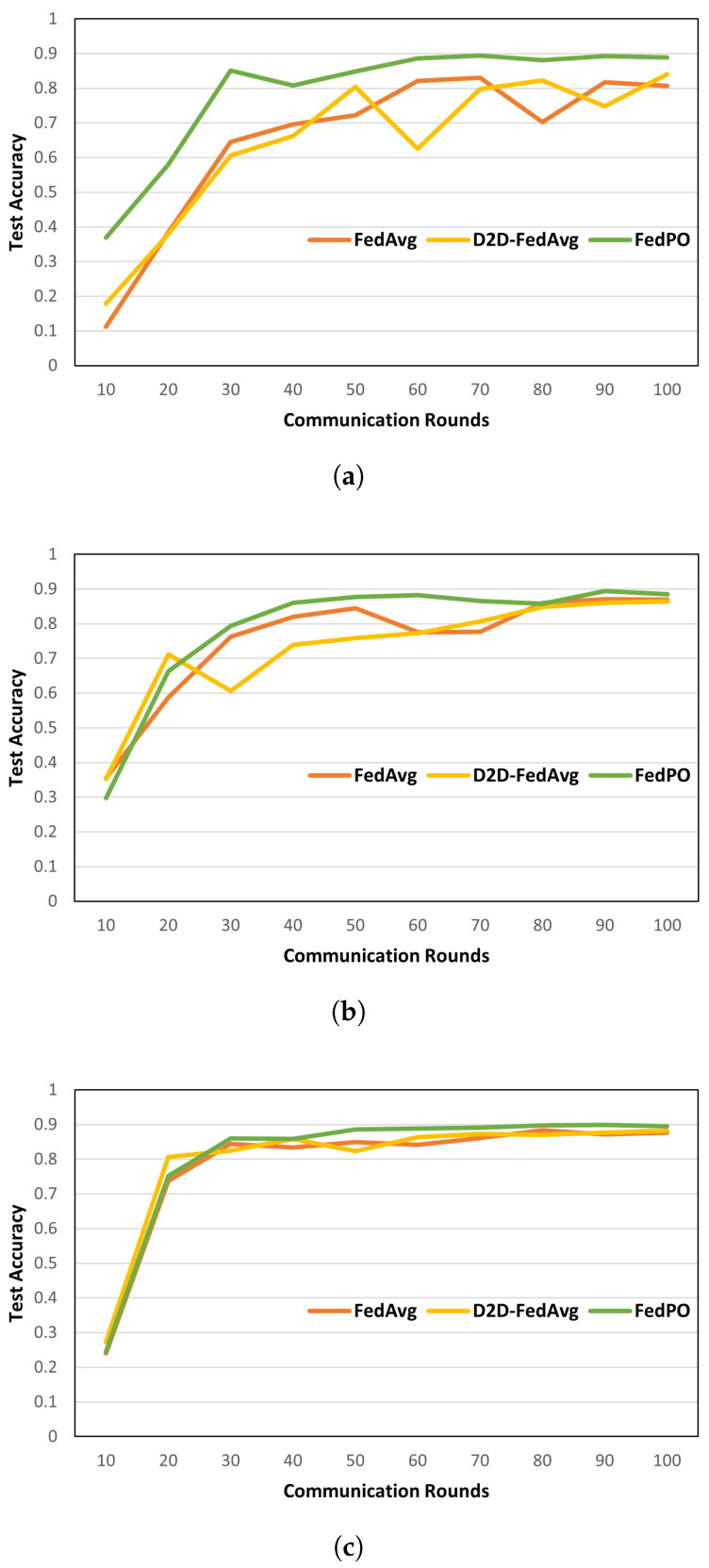
Model accuracy on MNIST for different values of prate with LR training model. (**a**) *prate* = 0.25. (**b**) *prate* = 0.50. (**c**) *prate* = 0.75.

**Figure 7:**
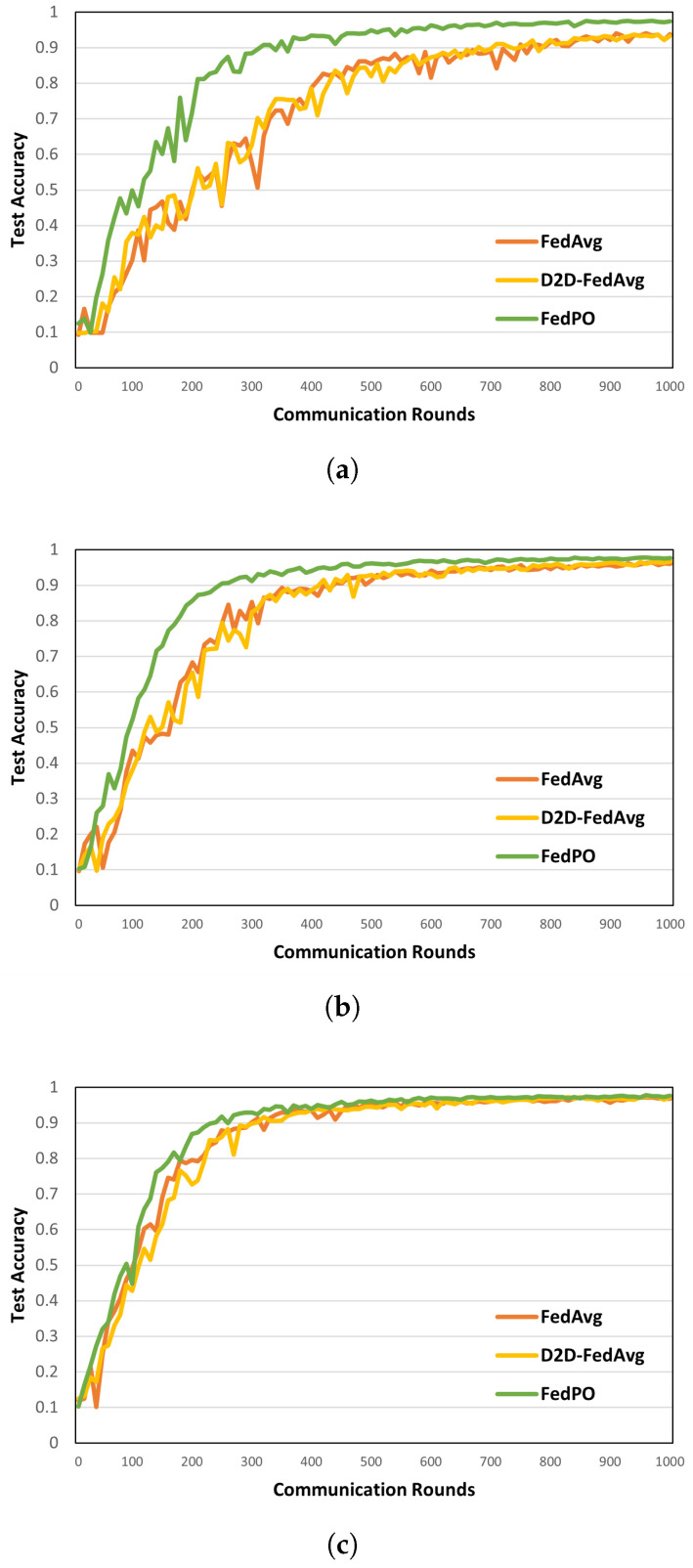
Model accuracy on MNIST for different values of prate with LSTM training model. (**a**) *prate* = 0.25. (**b**) *prate* = 0.50. (**c**) *prate* = 0.75.

**Figure 8:**
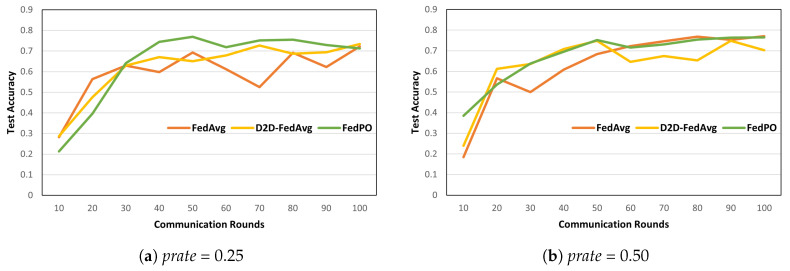

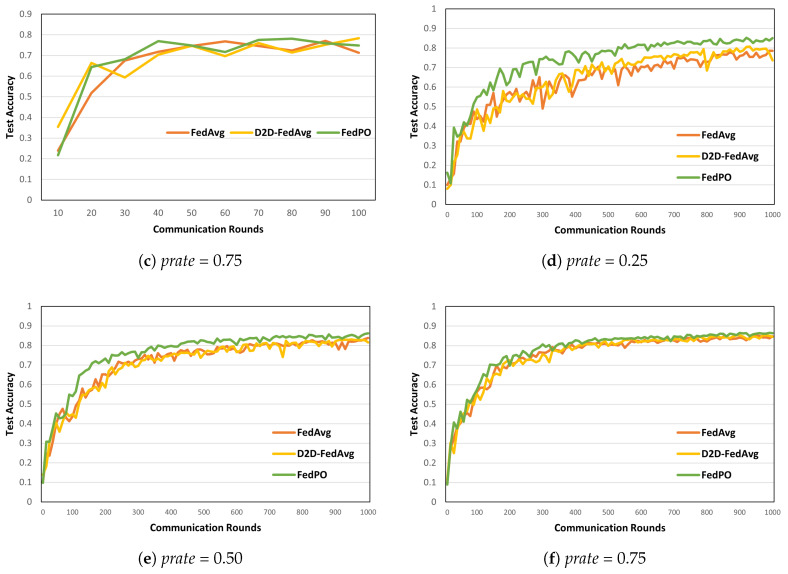
Model accuracy on FMNIST for different values of prate and learning models. (**a**–**c**) LR and (**d**–**f**) LSTM.

**Model Performance. **Figure 6 and Figure 7 show the model accuracies of FedAvg, D2D-FedAvg, and FedPO, where the LR and LSTM learning models are trained using the MNIST dataset with prate = 0.25, 0.50, and 0.75. We set a small number of clients (|K|=20) to clearly demonstrate that biased client selection is superior to unbiased client selection. Figure 6 shows the results of the first 100 rounds out of 1000 rounds to demonstrate the initial convergence with the LR training model. When prate = 0.25, the accuracy of the initial training differs by that *T* = 100 by 14%. For the remaining values of prate, similar model accuracy is obtained as the number of participating clients increases. Figure 7 shows the accuracy of the LSTM training model for *T* = 1000 rounds on the MNIST dataset. Unlike that for the LR, the effectiveness of FedPO is considerably higher than those of FedAvg and D2D-FedAvg when prate = 0.25 and 0.50. The results for FashionMNIST are shown in Figure 8. Additionally, the results show that FedPO achieves higher accuracy with fewer participating clients, particularly excelling in the case of LSTM models, similar to the results with MNIST.

## 6. Conclusions and Future Work

This paper proposes a new FL scheme named FedPO that uses k-means clustering for D2D communication and utilizes Pareto optimality to select participating clients based on their resource state and loss. The effectiveness of the proposed scheme is experimentally evaluated through experiments in comparison with two methods: FedAvg, the conventional centralized method, and D2D-FedAvg, a modified version for D2D communications.

Thus, FedPO is a promising approach for addressing bottlenecks, reducing server-side traffic, and saving client resources. Additionally, this method achieves faster model convergence in the initial rounds compared with the other methods.

In future work, additional experiments should be performed to evaluate the effect of environmental factors, such as communication instability and disconnection, on the FL performance. Furthermore, although we use Pareto optimality to select clients based on their loss and resource state, a wider range of considerations, such as battery life, connectivity, and computational capabilities of devices in real-world settings, may be considered for client selection. In addition, when selecting the threshold in k-means clustering, factors that may affect model convergence may be considered in addition to communication aspects.

Future research directions for FedPO implementation can be summarized as follows:

- Considering the effect of environmental factors on the FL performance: future work can be aimed at examining the effects of factors such as communication instability, network disconnection, and device heterogeneity on the FL performance.
- Optimizing the clustering approach: when selecting the threshold for k-means clustering, other factors affecting the model convergence, such as the data distribution and number of clusters, can be considered.
- Evaluating the performance of the proposed approach in real-world scenarios: The experiments in this study are conducted in simulated environments. In future work, the performance of the proposed approach can be evaluated in real-world settings to assess its practicality and effectiveness.

## Figures and Tables

**Table 1:** Summary of FL technique characteristics.

Method	Communication Method	Hierarchical Architecture	Client Selection
FedAvg [11]	central		
FedAsync [12]	central		
POWER-OF-CHOICE Strategy [14]	central		✓
FedCS [15]	central		✓
HFL [17,18,19]		✓	
D2D-assisted hierarchical FL [22]	D2D	✓	
SD-FEEL [23]	Edge Server		
TT-HF [24]	D2D		
P2P FL [26]	P2P		
FedPO	D2D	✓	✓

**Table 2:** Parameters for D2D network simulation.

Parameter	Value
Number of clients	100
Max. transmit power of the client, $P_{max}|dB$	23 dBm
Noise power level	−174 dBm/Hz
Transmit power of the parameter server	43 dBm
Maximum distance between LC and clients	200 m
threshold	

**Table 3:** Server-side traffic ratios of D2D-FedAvg, FedPO, and FedAvg.

Schemes	Prate	Transmitted (%)	Received (%)
FedAvg	0.25	100	28.12
	0.50	-	70.65
	0.75	-	100
D2D-FedAvg	0.25	23.57	20.94
	0.50	28.05	21.63
	0.75	37.99	22.37
FedPO	0.25	23.73	21.09
	0.50	23.67	21.23
	0.75	24.11	21.27

## Data Availability

The data presented in this study are available on request from the corresponding author.
